# Relationship Between Socioeconomic Status and the Prevalence of Underweight, Overweight or Obesity in a General Japanese Population: NIPPON DATA2010

**DOI:** 10.2188/jea.JE20170249

**Published:** 2018-03-05

**Authors:** Tomiyo Nakamura, Yasuyuki Nakamura, Shigeyuki Saitoh, Tomonori Okamura, Masahiko Yanagita, Katsushi Yoshita, Yoshikuni Kita, Yoshitaka Murakami, Hiroshi Yokomichi, Nobuo Nishi, Nagako Okuda, Aya Kadota, Takayoshi Ohkubo, Hirotsugu Ueshima, Akira Okayama, Katsuyuki Miura

**Affiliations:** 1 Department of Food Science and Human Nutrition, Ryukoku University, Shiga, Japan; 2 School of Health Sciences, Sapporo Medical University, Hokkaido, Japan; 3 Department of Preventive Medicine and Public Health, Keio University School of Medicine, Tokyo, Japan; 4 Faculty of Health and Sports Science, Doshisha University, Kyoto, Japan; 5 Department of Food and Human Health Science, Osaka City University Graduate School of Human Life Science, Osaka, Japan; 6 Faculty of Nursing Science, Tsuruga Nursing University, Fukui, Japan; 7 Department of Medical Statistics, School of Medicine, Toho University, Tokyo, Japan; 8 Department of Health Sciences, University of Yamanashi, Yamanashi, Japan; 9 International Center for Nutrition and Information, National Institute of Health and Nutrition, National Institutes of Biomedical Innovation, Health and Nutrition, Tokyo, Japan; 10 Department of Health and Nutrition, University of Human Arts and Sciences, Saitama, Japan; 11 Center for Epidemiologic Research in Asia, Shiga University of Medical Science, Shiga, Japan; 12 Department of Public Health, Shiga University of Medical Science, Shiga, Japan; 13 Department of Hygiene and Public Health, Teikyo University School of Medicine, Tokyo, Japan; 14 Research Institute of Strategy for Prevention, Tokyo, Japan

**Keywords:** socioeconomic status, overweight, obesity, education, household income

## Abstract

**Background:**

Socioeconomic status (SES) imbalances in developed and developing countries may result in individuals being overweight and obese. However, few studies have investigated this issue in Japan. We herein examined the relationship between SES and being underweight, overweight or obese according to sex and age groups (20–64 or ≥65 years) in Japan.

**Methods:**

In 2010, we established a cohort of participants in the National Health and Nutrition Survey of Japan. We divided 2,491 participants (1,081 men and 1,410 women) according to the WHO definitions of underweight, overweight or obesity and performed multinomial logistic analyses using BMI <18.5 kg/m^2^ (underweight), BMI 25.0–29.9 kg/m^2^ (overweight), and BMI ≥30.0 kg/m^2^ (obese) versus BMI 18.5–24.9 kg/m^2^ (normal) as the outcome, with SES groups as the main explanatory variables.

**Results:**

In adult men, a lower education level relative to a higher education level was inversely associated with obesity after adjustments for other SESs (odds ratio [OR] 0.41; 95% confidence interval [CI], 0.18–0.96). However, in adult women, lower education level was positively associated with being overweight and obese (OR 1.67; 95% CI, 1.07–2.49 for overweight and OR 2.66; 95% CI, 1.01–7.01 for obese). In adult women, a lower household income was positively associated with being overweight and obese (obese: OR 4.84; 95% CI, 1.36–17.18 for those with a household income <2 million JPY relative to those with ≥6 million JPY).

**Conclusions:**

In adult women, a lower education level and lower household income were positively associated with being overweight or obese. In contrast, in adult men, a lower education level was inversely associated with obesity. Gender and age differences in SESs affect the prevalence of being overweight or obese.

## INTRODUCTION

Being overweight and obese in combination with having cardiovascular metabolic risk factors^1^ is known to affect the incidence of and death from cardiovascular diseases. The World Health Organization (WHO) previously reported that more than 1.9 billion adults were overweight or obese (body mass index [BMI] ≥25.0 kg/m^2^), with more than 600 million being obese (BMI ≥30.0 kg/m^2^) worldwide in 2014.^2^ In a systematic analysis of population-based studies, the percentage of adults who were overweight or obese increased from 28.8% in 1980 to 36.9% in 2013 for men and from 29.8% to 38.0% for women.^3^ However, the increase in adult obesity in developed countries has slowed down since 2006.^3^

Among high-income countries, the average BMI in Japan was the lowest for men and women^2^; the percentage of those who were overweight or obese was 29.5% for men and 19.2% for women in 2015.^4^ Although there have been no significant changes in the percentage of men who are overweight or obese in the past 10 years, that of women has significantly decreased.^5^ The percentage of underweight women aged 20–29 years was 22.3%, while that of the elderly (older than 65 years) with malnutrition (BMI ≤20.0 kg/m^2^) was 16.7% in 2015.^4^ Therefore, not only being overweight or obese, but also being underweight (BMI <18.5 kg/m^2^) have the potential to become a public health issue. The percentages of individuals who are underweight, overweight or obese in Japan are unique from a global viewpoint, and vary depending on gender and generation.^4^^,^^5^

Previous studies suggested that socioeconomic status (SES) imbalances, such as education, occupation and income, in developed and developing countries were associated with being overweight or obese.^6^^–^^9^ These findings varied based on the developmental state of the countries examined.^6^ In countries with a low income or low human development index, a higher income/education level was associated with a greater prevalence of obesity in men and women.^7^ However, in middle-income countries, relationships between income/education levels and obesity were inconsistent.^7^ Furthermore, as gross national incomes and urbanization levels have increased, the prevalence of being overweight among women in the lowest education level has markedly increased,^8^ while a lower education level/occupation was associated with a higher BMI in women in highly developed countries.^9^

In Japan, SES imbalances are basically small, and few studies have examined the relationships among SES and being overweight or obese. One study used the findings from the Comprehensive Survey of Living Conditions (CSLC) and the National Health and Nutrition Survey of Japan (NHNS) in 2003–2007 and found relationships among a low equivalent household expenditure (EHE, monthly household expenditure divided by the square root of the number of household members), married status and obesity in women.^10^ NHNS2010 reported that the proportion of overweight or obese individuals in low-income households was higher in women, but not in men.^11^ This report did not consider other elements of SES, such as education, occupation, and marital and living statuses. In the present study, we examined relationships among these elements of SES and being underweight, overweight or obese according to sex and age groups.

## METHODS

### Study population

In 2010, a prospective cohort study on cardiovascular disease, the National Integrated Project for Prospective Observation of Non-communicable Disease and its Trends in the Aged 2010 (NIPPON DATA2010),^12^ was established. This study was performed using data from NHNS in November 2010 (NHNS2010) and CSLC in June 2010 (CSLC2010), which were conducted by the Ministry of Health, Labour and Welfare, Japan. The details of NHNS2010 and CSLC2010 have been described elsewhere.^13^^–^^17^

In November 2010, 8,815 residents aged 1 year and older from 300 randomly selected districts throughout Japan participated in NHNS2010. Among 7,229 participants aged 20 years and older, 3,873 (1,598 men and 2,275 women) who had a blood test were invited to enroll in the NIPPON DATA2010 Study. A total of 2,898 participants (1,239 men and 1,659 women) agreed to participate in the study via informed consent obtained by trained interviewers. They also agreed to an additional survey for NIPPON DATA2010, which included a questionnaire.

NHNS2010 participants were selected from participants for CSLC2010. Data obtained from NHNS2010 and CSLC2010 were merged with data from NIPPON DATA2010.^12^ Among the 2,898 participants for NIPPON DATA2010, 91 were excluded because it was not possible to merge the data from NHNS2010 or CSLC2010 with NIPPON DATA2010 baseline data, and 203 were excluded as maternal and lactating women or because data was missing on BMI, lifestyle, or SES. One hundred and thirteen participants who answered that he/she did not know the answer to SES questions were excluded from the analysis. The remaining 2,491 participants (1,081 men and 1,410 women) were included in the present study. The Institutional Review Board of Shiga University of Medical Science (No. 22-29, 2010) approved this study.

### Outcomes

The height and weight of participants were measured without shoes in NHNS2010. BMI was calculated by dividing weight in kilograms by height in meters squared. According to the WHO definition,^2^ participants were categorized as being underweight if BMI <18.5, normal if BMI was between 18.5 and 24.9, overweight if BMI was between 25.0 and 29.9 kg/m^2^, and obese if BMI was ≥30 kg/m^2^.

### Socioeconomic status (SES)

Information on SES was collected from questionnaires for NHNS2010 (type of occupation and annual household income), for CSLC2010 (number of household members, EHE) and for NIPPON DATA 2010 (education level, marital status [married or unmarried], and living alone/not alone). SES was defined as follows: size of the household (1, 2, ≥3), education level (to junior high school, junior college or higher), occupational status (employed [including self-employed] or unemployed [including students and homemakers]), marital and living statuses (married, single [including never married, divorced, and widowed] and not living alone, or single and living alone) and annual household income (<2,000,000 Japanese Yen [JPY], 2,000,000–6,000,000 JPY, or ≥6,000,000 JPY).

### Statistical analysis

In Japan, individuals aged 65 years or older are commonly defined as the elderly, and many retire before the age of 65. Therefore, all analyses were performed separately according to sex and two age groups: adults (20–64 years) and the elderly (≥65 years). Continuous data were described as means and standard deviations (SD), and analysis of variance followed by a post hoc application of Dunnett’s test when the *F* value showed a significant difference at *P* < 0.05. Categorical data were described as a number and percentage and were analyzed using χ^2^ tests. Multinomial logistic regression models was used to calculate odds ratios (ORs) and 95% confidence intervals (CIs) for the prevalence of being underweight (BM <18.5 kg/m^2^), overweight (BMI 25.0–29.9 kg/m^2^), or obese (BMI ≥30.0 kg/m^2^) versus normal (BMI 18.5–24.9 kg/m^2^), adjusting for age (per 5-year intervals) and the square root number of household members (only in the analysis for household income) in model 1; education level, marital and living statuses, household income, and the size of the household (square) were additionally adjusted in model 2. All reported *P* values are two-tailed. *P* values <0.05 were considered to be significant. All statistical analyses were performed using SAS version 9.4 for Windows (SAS Institute Inc., Cary, NC, USA).

## RESULTS

The mean age of participants was 60.0 years (range 20–91) in men, and 58.4 years (range 20–90) in women. The range of BMI was 14.7 to 41.3 kg/m^2^ in men, and 14.6 to 40.6 kg/m^2^ in women. The background characteristics of the participants according to sex, age, and BMI groups are presented in Table 1 and Table 2.

**Table 1.** Background characteristics according to BMI categories in men (NIPPON DATA2010)

Variables		BMI, kg/m^2^								*P* value

		≤18.5		18.6**–**24.9		25**–**29.9		≥30		
**Adult men (20–64 years)**										
*n* (%)		16	(2.7)	360	(61.4)	183	(31.2)	27	(4.6)	
Age, years		43.1	(14.9)	48.7	(12.3)	50.8	(10.8)	45.8	(9.3)	0.02
BMI, kg/m^2^		17.2	(0.9)	22.3	(1.8)	26.9	(1.4)	32.6	(2.6)	<0.01
Number of household members, *n* (%)	1	4	(25.0)	44	(12.2)	19	(10.4)	3	(11.1)	
	2	5	(31.3)	99	(27.5)	44	(24.0)	4	(14.8)	0.34
	≥3	7	(43.8)	217	(60.3)	120	(65.6)	20	(74.1)	
Education level, *n* (%)	Higher education	11	(68.8)	148	(41.1)	68	(37.2)	16	(59.3)	0.02
	Lower education	5	(31.3)	212	(58.9)	115	(62.8)	11	(40.7)	
Marital and living statuses, *n* (%)	Married	8	(50.0)	284	(78.9)	144	(78.7)	18	(66.7)	0.06
	Single, not living alone	4	(25.0)	42	(11.7)	24	(13.1)	7	(25.9)	
	Single, living alone	4	(25.0)	34	(9.4)	15	(8.2)	2	(7.4)	
Occupational status, *n* (%)	Employed	16	(100.0)	312	(41.1)	161	(37.2)	24	(59.3)	0.46
	Unemployed	—		48	(58.9)	22	(62.8)	3	(40.7)	
Household income, JPY, *n* (%)	≥6,000,000	4	(25.0)	103	(28.6)	50	(27.3)	5	(18.5)	
	2,000,000–6,000,000	9	(56.3)	210	(58.3)	101	(55.2)	18	(66.7)	0.76
	<2,000,000	3	(18.8)	47	(13.1)	32	(17.5)	4	(14.8)	
**Elderly men (≥65 years)**										
*n* (%)		20	(4.0)	323	(65.3)	146	(29.5)	6	(1.2)	
Age, years		77.1	(6.4)^**^	72.7	(5.9)	73.1	(5.7)	73.3	(4.8)	0.02
BMI, kg/m^2^		17.6	(0.7)	22.4	(1.7)	26.6	(1.2)	32	(2.9)	<0.01
Number of household members, *n* (%)	1	2	(10.0)	46	(14.2)	17	(11.6)	2	(33.3)	
	2	8	(40.0)	178	(55.1)	85	(58.2)	2	(33.3)	0.38
	≥3	10	(50.0)	99	(30.7)	44	(30.1)	2	(33.3)	
Education level, *n* (%)	Higher education	1	(5.0)	80	(24.8)	35	(24.0)	1	(16.7)	0.24
	Lower education	19	(95.0)	243	(75.2)	111	(76.0)	5	(83.3)	
Marital and living statuses, *n* (%)	Married	17	(85.0)	276	(85.4)	128	(87.7)	4	(66.7)	0.15
	Single, not living alone	2	(10.0)	12	(3.7)	9	(6.2)	—		
	Single, living alone	1	(5.0)	35	(10.8)	9	(6.2)	2	(33.3)	
Occupational status, *n* (%)	Employed	7	(35.0)	117	(36.2)	50	(34.3)	—		0.33
	Unemployed	13	(65.0)	206	(63.8)	96	(65.8)	6	(100.0)	
Household income, JPY, *n* (%)	≥6,000,000	3	(15.0)	44	(13.6)	19	(13.0)	—		
	2,000,000–6,000,000	13	(65.0)	199	(61.6)	99	(67.8)	3	(50.0)	0.55
	<2,000,000	4	(20.0)	80	(24.8)	28	(19.2)	3	(50.0)	

BMI, body mass index; JPY, Japanese yen; SD, standard deviation.

Data are presented as mean (SD) or as a number (%).

*P* values were calculated using a one-way analysis of variance for continuous variables or the chi-squared test for categorical variables.

^*^P < 0.05; ^**^P < 0.01, significantly different from the BMI group using Dunnett’s test.

**Table 2.** Background characteristics according to BMI categories in women (NIPPON DATA2010)

		BMI, kg/m^2^								*P* value^a^

		≤18.5		18.6**–**24.9		25**–**29.9		≥30		
**Adult women (20–64 years)**										
*n* (%)		92	(11.1)	571	(68.8)	141	(17.0)	26	(3.1)	
Age, years		42.4	(13.7)^**^	48.0	(11.2)	52.1	(10.8)^**^	50.0	(12.7)	<0.01
BMI, kg/m^2^		17.4	(0.7)	21.5	(1.8)	26.8	(1.3)	33.2	(2.6)	<0.01
Number of household members, *n* (%)	1	2	(2.2)	43	(7.5)	11	(7.8)	1	(3.9)	
	2	23	(25.0)	172	(30.1)	43	(30.5)	9	(34.6)	0.39
	≥3	67	(72.8)	356	(62.4)	87	(61.7)	16	(61.5)	
Education level, *n* (%)	Higher education	43	(46.7)	273	(47.8)	42	(29.8)	6	(23.1)	<0.01
	Lower education	49	(53.3)	298	(52.2)	99	(70.2)	20	(76.9)	
Marital and living statuses, *n* (%)	Married	63	(68.5)	455	(79.7)	114	(80.9)	19	(73.1)	0.18
	Single, not living alone	27	(29.3)	83	(14.5)	21	(14.9)	7	(26.9)	
	Single, living alone	2	(2.2)	33	(5.8)	6	(4.3)	—		
Occupational status, *n* (%)	Employed	51	(55.4)	344	(60.2)	99	(70.2)	17	(76.9)	0.18
	Unemployed	41	(44.6)	227	(39.8)	42	(29.8)	9	(23.1)	
Household income, JPY, *n* (%)	≥6,000,000	35	(38.0)	172	(30.1)	25	(17.7)	4	(15.4)	
	2,000,000**–**6,000,000	43	(46.7)	331	(58.0)	92	(65.3)	13	(50.0)	<0.01
	<2,000,000	14	(15.2)	68	(11.9)	24	(17.0)	9	(34.6)	
**Elderly women (≥65 years)**										
*n* (%)		35	(6.0)	387	(66.7)	133	(22.9)	25	(4.3)	
Age, years		74.5	(6.7)	73	(5.8)	73.1	(5.8)	74.6	(6.2)	0.33
BMI, kg/m^2^		17.3	(1.0)	22.1	(1.8)	26.7	(1.3)	31.5	(1.7)	<0.01
Number of household members, *n* (%)	1	7	(20.0)	101	(26.1)	36	(27.1)	11	(44.0)	
	2	14	(40.0)	186	(48.1)	62	(46.6)	6	(24.0)	0.02
	≥3	14	(40.0)	100	(25.8)	35	(26.3)	8	(32.0)	
Education level, *n* (%)	Higher education	5	(14.3)	65	(16.8)	29	(21.8)	4	(16.0)	0.43
	Lower education	30	(85.7)	322	(83.2)	104	(78.2)	21	(84.0)	
Marital and living statuses, *n* (%)	Married	22	(62.9)	258	(66.7)	82	(61.7)	10	(40.0)	0.18
	Single, not living alone	7	(20.0)	51	(13.2)	20	(15.0)	6	(24.0)	
	Single, living alone	6	(17.1)	78	(20.2)	31	(23.3)	9	(36.0)	
Occupational status, *n* (%)	Employed	5	(8.6)	65	(12.4)	29	(9.8)	4	(4.0)	0.56
	Unemployed	30	(91.4)	322	(87.6)	104	(90.2)	21	(96.0)	
Household income, JPY, *n* (%)	≥6,000,000	6	(17.1)	40	(10.3)	9	(6.8)	—		
	2,000,000**–**6,000,000	20	(57.1)	217	(56.1)	71	(53.4)	14	(56.0)	0.20
	<2,000,000	9	(25.7)	130	(33.6)	53	(39.8)	11	(44.0)	

BMI, body mass index; JPY, Japanese yen; SD, standard deviation.

Data are presented as mean (SD) or as a number (%).

^a^*P* values were calculated using a one-way analysis of variance for continuous variables or the chi-squared test for categorical variables.

^*^P < 0.05; ^**^P < 0.01, significantly different from the normal BMI group using Dunnett’s test.

In adult men, mean age significantly differed among BMI categories; however, post hoc analyses showed no significant differences in means from the normal BMI group. In elderly men, mean age was significantly older in the underweight group than in the normal BMI group (Table 1). Significant differences were observed in the education level among BMI categories in adult men. In adult women, mean age was significantly younger in the underweight group, but significantly older in the overweight group than in the normal BMI group (Table 2). Significant differences were observed in the education level and household income among BMI categories in adult women (Table 1).

The results of multinomial logistic regression analyses are shown in Table 3. In adult men, a lower education level relative to a higher education level was inversely associated with obesity after adjustments for other SESs (model 2 OR 0.41; 95% CI, 0.18–0.96). However, in adult women, it was positively associated with being overweight and obese (model 2 OR 1.67; 95% CI, 1.07–2.49 for overweight and OR 2.66; 95% CI, 1.01–7.01 for obesity).

**Table 3.** Socioeconomic status and odds ratios for being underweight, overweight or obese, NIPPON DATA2010 (multinomial logistic regression, *n* = 2,491)

Variables	underweight/normal				overweight/normal				obese/normal			
		
	OR1^a^	95% CI	OR2^b^	95% CI	OR1^a^	95% CI	OR2^b^	95% CI	OR1^a^	95% CI	OR2^b^	95% CI
**Adult men (20–64 years)**												
Education level												
Higher education	1.00		1.00		1.00		1.00		1.00		1.00	
Lower education	0.36	(0.12–1.07)	0.33	(0.10–1.05)	1.09	(0.75–1.58)	1.02	(0.69–1.51)	0.51	(0.23–1.14)	0.41	(0.18–0.96)^*^
Marital and living status												
Married	1.00		1.00		1.00		1.00		1.00		1.00	
Single, not living alone	2.37	(0.60–9.39)	2.75	(0.66–11.49)	1.44	(0.80–2.58)	1.59	(0.87–2.92)	2.37	(0.84–6.70)	2.61	(0.87–7.85)
Single, living alone	3.95	(1.12–13.93)^*^	3.75	(0.58–24.47)	0.89	(0.47–1.70)	1.20	(0.54–2.66)	0.92	(0.20–4.13)	1.35	(0.22–8.46)
Occupational status												
Employed	1.00		1.00		1.00		1.00		1.00		1.00	
Unemployed	—		—		0.78	(0.45–1.36)	0.67	(0.37–1.20)	0.93	(0.26–3.29)	0.71	(0.18–2.73)
Household income, JPY												
≥6,000,000	1.00		1.00		1.00		1.00		1.00		1.00	
2,000,000**–**6,000,000	0.90	(0.26–3.11)	1.10	(0.31–3.97)	1.03	(0.68–1.57)	1.03	(0.67–1.59)	1.85	(0.66–5.16)	2.19	(0.77–6.23)
<2,000,000	1.78	(0.36–8.85)	3.25	(0.57–18.58)	1.39	(0.78–2.50)	1.46	(0.77–2.74)	2.22	(0.55–8.92)	2.86	(0.64–12.88)
**Elderly men (≥65 years)**												
Education level												
Higher education	1.00		1.00		1.00		1.00		1.00		1.00	
Lower education	6.06	(0.80–46.21)	5.68	(0.72–44.69)	1.04	(0.66–1.64)	1.13	(0.71–1.81)	1.64	(0.19–14.25)	1.07	(0.11–10.22)
Marital and living statuses												
Married	1.00		1.00		1.00		1.00		1.00		1.00	
Single, not living alone	2.29	(0.45–11.61)	2.83	(0.54–14.91)	1.60	(0.66–3.91)	1.63	(0.66–4.01)	—		—	
Single, living alone	0.42	(0.05–3.31)	0.83	(0.09–8.09)	0.55	(0.26–1.19)	0.50	(0.21–1.17)	3.93	(0.69–22.27)	10.44	(0.89–122.63)
Occupational status												
Employed	1.00		1.00		1.00		1.00		1.00		1.00	
Unemployed	0.77	(0.29–2.06)	0.94	(0.34–2.61)	1.06	(0.70–1.62)	1.03	(0.67–1.58)	—		—	
Household income, JPY												
≥6,000,000	1.00		1.00		1.00		1.00		1.00		1.00	
2,000,000**–**6,000,000	1.72	(0.43–6.96)	1.80	(0.43–7.56)	1.12	(0.61–2.05)	1.09	(0.59–2.01)	—		—	
<2,000,000	1.56	(0.28–8.56)	1.35	(0.23–7.82)	0.76	(0.36–1.59)	0.76	(0.36–1.61)	—		—	
**Adult women (20–64 years)**												
Education level												
Higher education	1.00		1.00		1.00		1.00		1.00		1.00	
Lower education	1.37	(0.86–2.16)	1.37	(0.86–2.20)	1.82	(1.20–2.75)^**^	1.67	(1.07–2.49)^*^	3.00	(1.14–7.70)^*^	2.66	(1.01–7.01)^*^
Marital and living statuses												
Married	1.00		1.00		1.00		1.00		1.00		1.00	
Single, not living alone	1.72	(0.99–2.97)	1.86	(1.02–3.38)^*^	1.32	(0.77–2.26)	1.32	(0.75–2.34)	2.44	(0.96–6.23)	1.74	(0.62–4.87)
Single, living alone	0.41	(0.10–1.78)	0.44	(0.45–4.55)	0.72	(0.29–1.78)	0.78	(0.28–2.21)	—		—	
Occupational status												
Employed	1.00		1.00		1.00		1.00		1.00		1.00	
Unemployed	1.06	(0.39–2.90)	1.48	(0.92–3.37)	0.71	(0.43–1.17)	1.27	(0.86–1.87)	0.91	(0.30–2.82)	0.78	(0.33–1.86)
Household income, JPY												
≥6,000,000	1.00		1.00		1.00		1.00		1.00		1.00	
2,000,000**–**6,000,000	0.64	(0.39–1.05)	0.58	(0.35–0.97)^*^	1.90	(1.17–3.08)^**^	1.70	(1.04–2.79)^*^	1.74	(0.56–5.44)	1.44	(0.45–4.55)
<2,000,000	1.12	(0.56–2.26)	1.00	(0.47–2.09)	2.34	(1.23–4.45)^**^	2.09	(1.07–4.09)^*^	5.97	(1.74–20.52)^**^	4.84	(1.36–17.18)^*^
**Elderly women (≥65 years)**												
Education level												
Higher education	1.00		1.00		1.00		1.00		1.00		1.00	
Lower education	1.41	(0.41–4.81)	1.47	(0.42–5.11)	1.30	(0.68–2.49)	1.22	(0.63–2.37)	3.16	(0.42–23.94)	2.69	(0.34–21.05)
Marital and living statuses												
Married	1.00		1.00		1.00		1.00		1.00		1.00	
Single, not living alone	1.35	(0.52–3.47)	1.27	(0.48–3.37)	1.25	(0.69–2.26)	1.22	(0.67–2.21)	2.86	(0.95–8.55)	2.81	(0.91–8.69)
Single, living alone	0.76	(0.29–2.01)	1.08	(0.33–3.53)	1.27	(0.76–2.10)	1.16	(0.62–2.27)	2.80	(1.05–7.47)^*^	4.02	(1.10–14.63)^*^
Occupational status												
Employed	1.00		1.00		1.00		1.00		1.00		1.00	
Unemployed	1.06	(0.39–2.90)	1.12	(0.41–3.09)	0.71	(0.43–1.17)	0.71	(0.42–1.17)	0.91	(0.30–2.82)	0.94	(0.30–2.99)
Household income, JPY												
≥6,000,000	1.00		1.00		1.00		1.00		1.00		1.00	
2,000,000**–**6,000,000	0.65	(0.24–1.81)	0.67	(0.24–1.86)	1.48	(0.67–3.25)	1.51	(0.68–3.33)	—		—	
<2,000,000	0.52	(0.15–1.80)	0.50	(0.14–1.74)	1.83	(0.77–4.35)	1.79	(0.76–4.25)	—		—	

CI, confidence interval; JPY, Japanese yen; OR, odds ratio.

The results of a multinomial logistic regression analysis are shown.

^a^OR1: Odds ratio adjusted for age (per 5-y intervals), and the square root of the number of household members (only in the analysis for household income).

^b^OR2: Odds ratio adjusted for age (per 5-y intervals), education level, marital and living statuses, household income, and the square root of the number of household members.

^*^P < 0.05, ^**^P < 0.01.

In adult women, being single but not living alone, relative to those who were married, was positively associated with being underweight (model 2 OR 1.86; 95% CI, 1.02–3.38). In elderly women, being single and living alone relative to being married was positively associated with being obese (model 2 OR 4.02; 95% CI, 1.10–14.63). In adult men, model 1 OR showed that being single and living alone was positively associated with being underweight (OR 3.95; 95% CI, 1.12–13.93); however, significance disappeared after adjustments for other elements of SES (model 2 OR 3.75; 95% CI, 0.58–24.47).

In adult women, a lower household income was positively associated with being overweight and obese (overweight: model 2 OR 1.70; 95% CI, 1.04–2.79 for those with a household income of 2–6 million JPY; model 2 OR 2.09; 95% CI, 1.07–4.09 for those with a household income of <2 million JPY relative to those with ≥6 million JPY; obesity: model 2 OR 4.84; 95% CI, 1.36–17.18 for those with <2 million JPY relative to those with ≥6 million JPY). In contrast, a lower household income was inversely associated with being underweight (model 2 OR 0.58; 95% CI, 0.35–0.97 for those with a household income of 2–6 million JPY relative to those with ≥6 million JPY). In adult men, elderly men, and elderly women, no relationships were observed between household income and BMI groups. The results of analyses using EHE were similar to those using household income. No relationships were observed between occupational status and BMI groups for all genders and age categories.

## DISCUSSION

In the present study, using a dataset obtained from national surveys of a general Japanese population, gender and age differences were observed in the relationship of being underweight, overweight or obese with elements of SES. A lower education level was associated with a significantly higher prevalence of obesity among adult women, but with a significantly lower prevalence of obesity among adult men. A lower household income was clearly associated with an elevated prevalence of being overweight or obese in adult women but not in men.

Gender differences have been reported in the relationships among education and economic levels and the prevalence of being overweight or obese, both in foreign countries and in Japan. A review previously showed that a number of studies had found relationships among lower SESs, particularly a lower education level, and a larger BMI for women in highly developed countries. However, these relationships were not clear for men.^9^ A baseline survey for a Japanese cohort study reported that a higher education level was associated with a lower BMI for women but not men.^18^ Previous findings from a meta-analysis using a survey from European countries demonstrated that women with a lower education level were more likely to be overweight or obese than men.^19^ A study using data from national surveys in Japan showed a correlation between lower household expenditure and obesity in women but not in men.^10^ Our results on adult women were consistent with previous findings. In the present analysis, adult men with a lower education level were significantly less likely to be obese than those with a higher education level after adjustments for other elements of SES.

The adjusted OR of obesity were significantly higher among elderly women who were living alone than among those who were married. A cross-sectional study in Japan showed that men who exclusively ate alone, which may result in the skipping of meals, were more likely to be underweight than those who ate with others; however, this relationship is not clear in women.^20^ In the present study, the prevalence of obesity was very small in elderly men, and we did not find a significant association in these participants. Eating alone and living alone may affect the prevalence of obesity.

We found no relationships between occupational status (employed/unemployed) and BMI groups. Factors that affect BMI (eg, labor intensity, wages, food access, and education level) may vary among job categories. Therefore, the relationships among job categories and BMI groups need to be examined in more detail.

We found several gender differences in the relationships among elements of SES and BMI categories. In men, living alone was associated with being underweight, and a lower education level was inversely associated with obesity. In women, lower education level, lower household income, and living alone were associated with being overweight/obese. Differences in basic living abilities for food preparation between men and women may have resulted in the differences observed in this relationship.

The strengths of this study were that we investigated a randomly selected sample of a general population of Japan. In addition, we examined relationships among elements of SES and each BMI category after adjustments for confounding factors.

The present study has several limitations. Since this was a cross-sectional study, we were unable to clarify whether there was a causal relationship between SES and the prevalence of being underweight, overweight or obese. A second limitation is the possibility that the influence of age was not well adjusted. Since aspects of SES varied widely with age, we analyzed elements of SES in three categories. However, due to the small number in each category, there was not enough power to test that relationship. Therefore, we analyzed two age groups depending on the age at which SES is most likely to change. Furthermore, we obtained information on household incomes instead of each participant’s income, for which we adjusted for the square root number of household members. However, it may not reflect each individual’s income, so the adjustment may not be sufficient.

In conclusion, the prevalence of being overweight or obese was significantly higher among adult women with a lower education level or household income. On the other hand, the prevalence of obesity was lower among adult men with a lower education level. Gender and age differences in the elements of SES affect not only the prevalence of being overweight and obese, but also being underweight in men and women.
